# An alternative source of elevated tau phosphorylation in plasma following renal dysfunction

**DOI:** 10.1002/alz70856_099292

**Published:** 2025-12-25

**Authors:** Tatsuya Manabe, Takuto Iida, Takashi Saito

**Affiliations:** ^1^ Nagoya City University, Nagoya, Aichi, Japan

## Abstract

**Background:**

Build‐ups of β‐amyloid (Aβ) in the brain represent the earliest pathological signatures of Alzheimer's disease (AD). Recent studies have spotlighted phosphorylated tau protein (*p*‐tau) as a biomarker for the Aβ burden. Notably, tau phosphorylated at the 181st and 217th threonine residues — *p*‐tau181 and *p*‐tau217 — accumulate in the blood from the preclinical AD. However, other age‐related diseases, such as chronic kidney disease (CKD), can increase *p*‐tau levels, irrespective of the amyloid pathology. To deepen our understanding of how circulating *p*‐tau could be enriched in those patients with CKD, we aim to clarify whether the elevated *p*‐tau can be caused by the increased *p*‐tau in the brain using the relevant mouse model.

**Method:**

To induce CKD, 9‐ and 18‐month‐old C57BL/6 mice were fed a 0.2% adenine‐rich diet for four weeks and the control diet for the next two weeks. Renal functions were assessed by picrosirius red staining and other biochemical analyses of the kidney, plasma, and urine. Tau phosphorylation in the brain was evaluated by western blot, while the three‐dimensional morphological analysis of microglia quantified neuroinflammatory responses to CKD.

**Result:**

Adenine caused renal fibrosis, as evidenced by collagen deposition and an eight‐fold rise of α‐smooth muscle actin in the kidneys of all the adenine‐fed mice. Plasma uremic toxins also significantly increased from the baseline if treated with adenine, and these changes were associated with the age of animals. Our analysis of brain tau phosphorylation identified that *p*‐tau181, *p*‐tau217, and *p*‐tau396 were heightened in the aged CKD mice. Similarly, microglia became less ramified if adenine was treated with the aged mice.

**Conclusion:**

Our data indicated that adenine recapitulated the CKD pathology over six weeks. Significant age‐related differences suggested (i) a delayed resolution of renal functions after the treatment or (ii) a more severe renal dysfunction in the aged mice. Altered *p*‐tau status and neuroinflammation onset in the aged CKD mouse brains underscored a novel kidney‐brain axis in CKD among elderly patients. Further investigation will concentrate on the chronic effect of CKD on *p*‐tau and whether circulating *p*‐tau levels could be affected in cerebrospinal fluid and plasma.

